# Health-related quality of life and long-term care needs among elderly individuals living alone: a cross-sectional study in rural areas of Shaanxi Province, China

**DOI:** 10.1186/1471-2458-13-313

**Published:** 2013-04-08

**Authors:** Ning Liu, Lingxia Zeng, Zhe Li, Jue Wang

**Affiliations:** 1 The Key Laboratory of Biomedical Information Engineering of Ministry of Education, and Research Center of Rehabilitation Science and Technology, School of Life Science and Technology, Xi’an Jiaotong University; National Engineering Research Center of Health Care and Medical Devices; Xi’an Jiaotong University Branch, Xi’an, , 710049, P.R. China; 2 Department of Public Health, College of Medicine, Xi’an Jiaotong University, Xi’an, Shaanxi Province, 710061, P.R. China; 3 Shaanxi Branch of China Telecom, Xi’an, Shaanxi Province, 710065, P.R. China

**Keywords:** Health-related quality of life, SF-36, Long-term care, Elderly subjects living alone

## Abstract

**Background:**

The number of elderly individuals living alone is rising, especially in rural areas of China, and their health-related quality of life (HRQoL) is an increasing public health concern. However, little is known about factors that influence HRQoL and the need for long-term care services. The aim of the study was to identify these factors and the long-term care requirements of persons aged 60 and older living alone in rural areas of Shaanxi Province, China.

**Methods:**

The study included 424 older subjects, selected by stratified random sampling. Logistic regression adjusted for age was conducted to analyze factors influencing HRQoL and the need for long-term care services. Pearson correlative analysis was conducted to assess the correlation between HRQoL score and long-term care needs.

**Results:**

HRQoL among elderly subjects living alone declined with age in both males and females. The main diseases influencing HRQoL among the elderly were hypertension, cardiac disease, chronic bronchitis, neurological disease and cancer. Cataract disease was the most important factor related to HRQoL. This was followed by long-term care needs, living conditions, economic status, Cardiovascular disease, osteoporosis and age. Factors affecting long-term care needs were economic status, education level, alcohol intake, living conditions, general health and age. HRQoL and long-term care needs among this elderly population were significantly correlated (r=−0.204, *p*<0.01).

**Conclusions:**

For elderly persons living alone, factors such as chronic disease, lower income level and living in a rural area may limit their ability to form social relationships. Reducing the level of loneliness, with better care and support, may be helpful in improving their HRQoL. There is a need for an overall improvement in the planning, provision and financing of long-term care and psychogeriatric services for elderly individuals living alone in China.

## Background

The proportion of older adults is increasing in most developing countries, and problems related to the rising number of these individuals living alone has become an important concern internationally [1]. As a result of China’s overall economic environment and the accelerated process of urbanization, the number of elderly individuals living alone is increasing, especially in some rural districts [2]. Rural elderly people often live in worse socioeconomic conditions and have poorer literacy skills compared with their urban counterparts [3]. Therefore, their social and physical wellbeing has become a challenging issue in China. A population-based study in Finland found that more than one-third of the aged (39.4%) suffered from loneliness [4]. In Italy, where the primacy of family support of the elderly has been decreasing in recent years, a low rate of interaction with friends was found to be associated with a decline in HRQoL, as measured by mental and physical health indicators [5]. Traditional Chinese culture, which emphasizes filial devotion, appears unprepared for a rapid growth in the number of empty-nest households. China will have to institute very rapid reforms to care for an increasingly older society, given its relatively limited social security system. Previous studies have developed patient-report measures for exploring QoL [6-10]. Many Chinese researchers have explored indicators relating to empty-nesters [2,11-13], including HRQoL. Li et al. [14] examined the association between living arrangements and health among very elderly Chinese and found that having a spouse in the household provided the best health protection, while living alone and living with children were associated with both health advantages and disadvantages. Feng and Chen [15] found that in Xiamen City, elderly subjects living alone had lower scores on a range of indicators relating to physical and mental health and social interaction, and older adults living alone had significantly worse QoL than those living with a family member. However, little is known about whether these findings are the same for older people, especially empty-nest individuals, living in rural areas of China. This study set out to determine the influences on HRQoL and the need for long-term care services of subjects aged 60 and older in rural areas of Shaanxi Province living at home alone without family.

## Methods

### Location

This was a cross-sectional survey of a random sample of older adults selected from three regions in rural areas of Shaanxi Province (Central Shaanxi, South Shaanxi and North Shaanxi) using a stratified, multiple-stage sampling method. These three regions are not only representative of Shaanxi with respect to geographical scale and distribution, but also represent the various types of industry in Shaanxi. Central Shaanxi is typical of well-developed regions and has 32 counties; North Shaanxi is representative of moderately developed regions and has 23 counties; and South Shaanxi is representative of underdeveloped regions and has 25 counties. These three regions are representative of the population with respect to lifestyle and geographical distribution. Therefore, a survey of older adults from these regions could well represent the HRQoL status of the elderly in Shaanxi.

### Subjects

The entire Shaanxi Province was broken down into three stratums: South Shaanxi, North Shaanxi and Central Shaanxi; and in each stratum, one county was completely randomly selected for the survey. The three counties were Ansai in North Shaanxi, Gaoling in Central Shaanxi and Shiquan in South Shaanxi. Participants in the present study were recruited from Ansai, Gaoling and Shiquan administrative areas by a cluster randomization method. The cohort for this survey was based on the local authority register for individuals born before 1950. Eligibility criteria were (i) residence in one of the three regions; (ii) age 60 or older and (iii) living alone and not with any children. Participants answered questions concerning sociodemographics, physical and mental health and their long-term care needs. If the individuals had limited autonomy, such as physical limitations or mental incapacity, their close relatives or neighbors could act as their proxy in accepting the invitation and helping to complete the questionnaires. In the target districts during the time of the study, all individuals’ records were obtained from the Sixth Nationwide Census, and all eligible individuals were invited to participate in the study.

### Instruments

The following instruments were used: (1) Demographic researcher-developed questionnaire measuring sociodemographic factors, including sex, age, marital status, yearly income, educational level and chronic diseases (see Additional file 1). (2) Short Form 36 Health Survey Questionnaire (SF-36). Although the SF-36 is American in origin, the Chinese version has good reliability and validity, and is appropriate for the evaluation of HRQoL in an elderly Chinese population [16]. The SF-36 consists of 36 items representing eight generic health concepts: physical functioning (PF) (10 items); social functioning (SF) (two items); role limitations due to physical problems (RP) (four items); role limitations due to emotional problems (RE) (three items); mental health (MH) (five items); vitality (VT) (four items); bodily pain (BP) (two items); and general perception of health (GH) (five items). Higher raw scores indicate better health. Subscale scores were combined and transformed into scores of 0 to 100 for physical and mental functioning, and presented as the mental component summary scale (MCS) and physical component summary scale (PCS) (see Additional file 2). (3) Care requirements were also ascertained including whether the individuals needed hospital-based care, community-based geriatric care or nursing home care. A questionnaire comprising five questions on participants’ long-term care needs. Each question could be answered “yes” or “no”. This questionnaire was developed using the Delphi expert consultation method (see Additional file 3).

### Quality control

Subjects were contacted first by letter and then by follow-up telephone call to explain the study. After giving informed consent, the subjects’ ages were confirmed using the household registration system. Approval of the village council was sought, accompanied by cooperation of the town health center. During the face-to-face field survey, trained research assistants explained how to fill in the questionnaires and helped participants complete them in their home, with the procedure lasting 30 minutes on average. The survey was conducted between August 2010 and February 2012. Questionnaires that were less than 80% complete were rejected. The database was established by EpiData3.02 (EpiData Association, Odense, Denmark), and double input was conducted to ensure accuracy.

### Data analysis

Data were analyzed using SPSS for Windows version 13.0 (SPSS Inc., Chicago, IL, USA). Clinical and sociodemographic variables were expressed as percentages, frequencies and means (± standard deviation). A logistic regression model was used to determine which chronic diseases affected the older adults’ HRQoL, the factors affecting HRQoL and long-term care needs with sex, age, chronic diseases, education level, living conditions and economic status as candidate factors. Pearson correlative analysis was conducted to assess the association between HRQoL score and long-term care needs. Principal component analysis of health indicators in the questionnaire was performed. Then, the first main component was assigned a value of 0 or 1 (greater than 0 for 1 and less than 0 for 0). Finally, those variables with a significant association were included as dependent variables in the logistic regression analysis. In the data analysis, we adjusted for the effect of different respondents on the results.

### Ethical considerations

The Ethics Committee of Xi’an JiaoTong University approved the study (NO. 2010006), and the research proposal was submitted to and approved by the Ethical Review Committee in the target region. Oral informed consent was obtained from each participant. Participants were assured of their right to refuse to participate or to withdraw from the study at any time. Anonymity and confidentiality of the participants were assured. Participants were presented with a small gift (valued 2.5 USD) on completion of the survey.

## Results

### Participants

**Figure 1:**
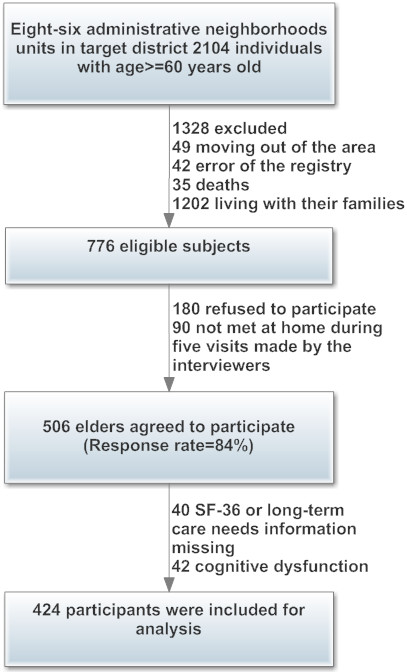
Flowchart of recruitment procedures in the current study.

In 86 administrative neighborhoods of the target country towns, there were 2,104 individuals aged 60 and older. We identified 1,328 who were not eligible (1,202 living with families, 35 had died, 49 had moved out of the area and 42 had errors in the registry). Of the remaining 776 individuals, 506 agreed to participate and provide information. Five questionnaires with more than 10 items not answered were not included in the data analysis. A total of 424 (84%) answered the questionnaires completely, and their data were analyzed. The proportion of missing data from each dimension of the SF-36 was low (0.5–4%). Because few data were missing for the SF-36 dimensions and the study sample was large, we did not substitute for missing data (see Figure 1).

Table 1 presents sociodemographic data and comorbid conditions (International Classification of Diseases, 9th Revision). The age range of the participants was between 60 and 80 years, with a mean of 70 years. Of the participants, 73.1% were female, 67.5% were illiterate and 28.5% had primary education only. In addition, 81.1% of participants had married, and 95.9% were farmers. The numbers of elderly living alone in Central China, South and North Shaanxi were 137 (32.3%), 146 (34.4%) and 141 (33.3%), respectively. The most common diseases were related to the cardiovascular system, with hypertension (58.3%) and cardiac disease (28.4%) being most prevalent.

**Table 1:** Sociodemographic characteristics and chronic diseases of elderly subjects living alone (n=424)

**Factors**	**Groups**	**Participants**	**Percent**
		**(n=424)**	**(%)**
Sex	Female	301	73.1
	Male	109	26.0
Age	60-64	169	40.1
	65-69	119	28.1
	70-74	73	17.2
	>=75	61	14.6
Site	Central Shaanxi Province	137	32.3
	South Shaanxi Province	146	34.4
	North Shaanxi Province	141	33.3
Education	Illiterate	286	67.5
	Primary	121	28.5
	Secondary	13	3.1
	University	4	0.9
Marital Status	Married	344	81.1
	Divorced	80	18.9
Economic Status	Poor	266	62.7
	Intermediate	129	30.4
	Good	29	6.8
Occupation	Farmer	407	95.9.
	No work	17	4.0
Chronic Disease	Hypertension	246	58.3
	Cardiac disease	120	28.4
	Endocrinological	47	11.1
	Gastrointestinal disease	65	15.4
	Orthopedic disease	151	35.9
	Oncological disease	47	11.1
	Other chronic diseases	96	22.7
	Neurological disease	125	29.6
	Chronic bronchitis	56	15.6
	Cataract disease	28	6.6
	Urinary disorders	27	6.4

### Evaluations of reliability and validity

Cronbach's α, a reflection of reliability of the long-term care needs questionnaire, exceeded 0.7 for all items (α = 0.866). Set validity and discrimination validity were calculated for each of the dimensions of the questionnaire. The relevant coefficient of each item, after removing the overlapping part of the dimension, was higher than 0.40. The relevance between each item within the dimension was significantly higher than the relevance between each item in other dimensions, suggesting that aggregate validity and discriminant validity were good [17].

### HRQoL of elderly subjects living alone in Shaanxi province

Table 2 shows that the HRQoL scores varied from 19.2 (± 39.1) to 82.6 (± 17.7). The RP and RE scores were lowest among the eight dimensions of the SF-36, with PF score being the highest. For PF, the subjects expressed difficulty in understanding the concept of walking distance. Although “mile” had been replaced in the Chinese version of the SF-36 by “kilometer”, the rural residents tended to interpret it as the Chinese measure “Li”, which means “0.5 kilometer”. The elderly in this study scored relatively higher on PF compared with the original US validation sample, which is consistent with reports by rural elderly adults that walking is considered one of the easiest activities in their daily lives. Regarding items measuring “accomplishment” in RP and RE, subjects explained that the demands of their job as a farmer varied. During quiet periods, they often were unoccupied, whereas during busy seasons, they felt they had to accomplish whatever was necessary regardless of their level of motivation.

**Table 2:** HRQoL of elderly subjects living alone in Shaanxi Province (n=424)

**Short Form-36**	**Min**	**Max**	**HRQoL (mean±SD)**
PF (physical functioning)	20	100	82.6 ± 17.7
RP (role functioning physical)	0	100	19.2 ± 39.1
BP (bodily pain)	0	70	31.9 ± 18.4
GH (general health perceptions)	25	70	54.7 ± 4.7
VT (vitality)	30	70	54.8 ± 6.8
SF (social functioning)	−12.5	87.5	40.7 ± 19.3
RE (emotional role functioning)	0	100	25.7 ± 43.4
MH (mental health)	24	56	41.8 ± 5.2

SD: Standard deviation.

### Chronic diseases affecting subjects’ HRQoL

Multiple regression was conducted to determine which chronic diseases affected the subjects’ HRQoL. After adjustment for age, we identified five diseases in the model: hypertension, cardiac disease, neurological disease, chronic bronchitis and cancer. The results are shown in Table 3.

**Table 3:** Association of chronic diseases with HRQoL among elderly subjects living alone

**Variable**	**B**	***t*-value**	***p*-value**	**OR**
Hypertension	−0.773	9.166	0.002	0.48
Cardiac disease	−0.889	8.641	0.003	0.41
Chronic bronchitis	1.929	5.141	0.023	0.31
Neurological disease	−0.761	6.918	0.009	0.45
Cancer	−4.09	15.702	<0.001	0.02

OR: odds ratio.

### Factors influencing HRQoL among elderly subjects living alone

Logistic regression was conducted to determine the factors affecting HRQoL. Seven variables were included in the regression model. Cataract disease was the most important factor related to HRQoL. This was followed by long-term care needs, living conditions, economic status, Cardiovascular disease and osteoporosis (see Table 4). Chronic diseases had a major impact on the HRQoL of rural elderly subjects. Cataract disease and cardiovascular disease remains an important disease affecting the quality of life of the elderly in rural areas of China.

**Table 4:** Factors influencing HRQoL among elderly subjects living alone

**Variables**	**B**	**SE**	**Waldχ**^**2**^	***p***	**OR**	**95% CI**
Age	−0.118	0.031	14.346	<0.001	0.89	0.84–0.95
Economic status	−0.908	0.195	21.659	0.006	0.40	0.28–0.59
Cardiovascular disease	−0.868	0.377	5.303	0.021	0.42	0.20–0.88
Living conditions	2.018	0.939	4.618	0.032	7.53	1.19–47.43
Osteoporosis	−0.757	0.367	4.264	0.039	0.47	0.23–0.96
Cataract disease	−3.159	1.104	8.193	0.004	0.04	0.01–0.37
Long-term care need	3.023	0.335	81.534	<0.001	20.67	10.67–39.62

SE: standard error; CI: confidence interval.

### Factors influencing long-term care needs among elderly subjects living alone

Logistic regression was conducted to analyze the factors influencing care needs of the elderly. The results showed that economic status was the largest contributor to long-term care needs, followed by education level, alcohol intake living conditions, GH and age (see Table 5). HRQoL and long-term care needs among this elderly population were significantly correlated (r =−0.204, p<0.01) (see Table 6).

**Table 5:** Factors affecting long-term care needs among elderly subjects living alone (n=424)

**Variables**	**B**	**SE**	**Wald χ**^**2**^	***p***	**OR**	**95% CI**
Age	−0.062	0.021	8.908	0.003	0.94	0.90–0.99
Education level	0.574	0.257	5.000	0.025	1.78	1.02–2.72
Living conditions	0.226	0.088	6.652	0.01	1.25	1.09–1.54
Economic status	−0.579	0.135	18.331	<0.001	0.56	0.43–0.73
Alcohol intake	−0.494	0.175	7.968	0.005	0.61	0.45–0.89
GH	−0.146	0.032	21.099	<0.001	0.86	0.84–0.94

**Table 6:** HRQoL among elderly subjects living alone according to age

**Age**	**Participants (n=424)**	**HRQoL score (Mean±SD)**
60-64	169	374.0±79.8
65-69	119	357.2±77.5
70-74	73	322.0±70.4
>=75	61	318.8±69.3

F=14.205, p<0.01.

## Discussion

Our results showed that frailty in older subjects living alone had marked negative effects on the eight dimensions of the SF-36. Managing elderly people with chronic disease is complex because they commonly have multiple chronic conditions. Effective chronic disease management requires recognition of this complexity because there may be conflicts among management guidelines for the multiple conditions present [18]. Older adults in rural areas of Shaanxi Province often have difficult pressures in life, including chronic diseases, ‘empty nest syndrome’, mental and physical disorders, serious psychological fatigue and psychological problems. The mental and physical disorders may in turn increase stress and lead to poor health and chronic diseases [19]. In the present study, hypertension,,cardiac disease, chronic bronchitis, neurological disease and cancer were the major conditions that impacted HRQoL, and, in turn, the need for long-term care services. Elderly subjects living alone may have lower social status and income as well as depression related to their feelings of isolation and loneliness, compounded by the rural location of farms, the nature of farm work and children living away from home, which may partially explain the socioeconomic inequalities in health [2,20]. This study indicated that we should focus on depression in the elderly, especially the empty-nest elderly in rural areas, to formulate effective measures to improve QoL. Chronic diseases were also associated with lower QoL, and perceived QoL was significantly correlated with self-rated health [21,22].

In accordance with earlier studies [23,24], education was a significant positive contributor to overall HRQoL. According to Lasheras et al. [25], lower educational level is associated with unhappiness, poor social relationships, poor self-assessed health and sensory problems among the elderly. Education is an important indicator that may directly or indirectly influence HRQoL through its association with higher social class and economic status. Our survey suggests that education level is associated with better HRQoL and a lower need for long-term care services among empty-nest elderly subjects. This is consistent with other studies conducted in Nigeria and Iran [23,26]. Where older adults with better education generally enjoy higher incomes and better social support. Medical costs are a huge burden for elderly subjects living alone because there is no universal health insurance in China, and may have a greater impact than in developed countries. Both education and income have a major influence on the QoL of the elderly in China. In our study, subjects reported higher physical functioning compared with perceived physical and emotional limitations, as reflected by scores on the SF-36. Furthermore, elderly people being able to live with their families is very important to health outcomes and has a positive effect on QoL [27]. Good social relationships (including relationships with relatives and friends) are the most commonly reported factor influencing QoL in the elderly [26,28,29]. There are fewer nursing homes in China than in developed countries. Policy-makers and health care providers should plan for the long-term care and health care delivery needs of the elderly, given that the placement of older adults in nursing homes is likely to increase in the coming years. Nursing homes will need to pay greater attention to the treatment of chronic diseases in older adults to improve their QoL. China has realized that expanding this area is not financially sustainable using only the government's resources. The current policy is to encourage private and foreign investors to participate in the nursing home business in China.

Our use of data from three regions in Shaanxi Province limited the applicability of our results to other provinces in China. Despite this, the results of the analysis provide an overall picture of the HRQoL and long-term care needs among older adults in rural Shaanxi Province, which may facilitate further prospective studies.

## Conclusions

In conclusion, a population of elderly individuals living alone in their homes in a rural area of China had low health care service utilization and generally poor HRQoL. Health authorities in China and community health centers need to provide these older people with adequate interventions, such as health education, health promotion and health resources. The government should encourage family members to provide more support for the elderly, and to take action to improve their care services. This study emphasizes the importance of support, and improved medical, health and counseling services for this group. It is recommended that all relevant stakeholders prioritize health promotion programs for the elderly and allocation of resources. In doing so, QoL among elderly citizens living alone in China can be improved.

## Abbreviations

HRQoL: Health-related quality of life; SF-36: Short form (36) Health Survey; PF: Physical Functioning; RP: Role-Physical; BP: Bodily Pain; GH: General Health; VT: Vitality; SF: Social Functioning; RE: Role-Emotional; MH: Mental Health.

## Competing interests

The authors declare that they have no competing interests.

## Authors’ contributions

NL and ZL developed the questionnaire and study design, supervised the analysis and contributed to the final version of the manuscript. JW, LZ and ZL assisted with the survey and data analyses and are the principal authors of this paper. All authors have read and approved the final manuscript.

## Pre-publication history

The pre-publication history for this paper can be accessed here:

http://www.biomedcentral.com/1471-2458/13/313/prepub

## Supplementary Material

Additional file 1 Subjects’ sociodemographic characteristics and chronic diseases. Click here for file

Additional file 2 The Medical Outcomes Study 36-Item Short-Form Health Survey. Click here for file

Additional file 3 Long-term care needs among elderly living alone. Click here for file
