# Supplementary material for: Health-related quality of life and long-term care needs among elderly individuals living alone: a cross-sectional study in rural areas of Shaanxi Province, China
Source: BMC Public Health. 2013 Apr 8;13:313. doi: 10.1186/1471-2458-13-313 (PMC3642010; doi:10.1186/1471-2458-13-313)
Supplement: Additional file 1 — Subjects’ sociodemographic characteristics and chronic diseases. [file 1471-2458-13-313-S1.doc]

## Additional File 1:Sociodemographic characteristics and their chronic diseases

In order to understand Health-related quality of life and long-term care needs among elderly subjects living alone in rural areas of Shaanxi Province, China. Provide a reference for the formation of China's health care plan for the elderly subjects living alone and the development of effective measures. We carry out the questionnaire, (Includes three parts) the information collected in the survey is entirely for academic research and confidential for the content of your answer. Please answer your actual situation, this research is very valuable to us, and hope to get your earnest cooperation. Please answer each question, choose the one that best suits their situation the answer, painted in the appropriate answer '√' or fill in value according to the actual situation. Participants were assured of their right to refuse to participate or to withdraw from the study at any time. Participants were presented with a small gift (valued 2.5 USD) on completion of the survey. Thank you very much.

(Research Center of Rehabilitation Science and Technology, School of Life Science and Technology, Xi'an Jiaotong University, Xi’an).

1. Sex: 1. Female 2.Male

2. Age: ____.

3. Site: 1. North Shaanxi Province(Ansai) 2. Central Shaanxi Province(Gaoling)

3. South Shaanxi Province(Shiquan).

4. Education: 1.Illiterate 2. Primary 3. Secondary 4. University.

5. Marital Status: 1. Married 2. Divorced.

6. Economic Status: 1. Poor 2. Intermediate 3, Good.

7,Yearly income: ____.

8. Occupation: 1. Farmer 2. No work.

9. Chronic Disease:

| Hypertension | 1. yes 2.no |
| --- | --- |
| Cardiac disease | 1. yes 2.no |
| Endocrinological | 1. yes 2.no |
| Gastrointestinal | 1. yes 2.no |
| Orthopedic disease | 1. yes 2.no |
| Oncological disease | 1. yes 2.no |
| Neurological disease | 1. yes 2.no |
| Chronic bronchitis | 1. yes 2.no |
| Cataract | 1. yes 2.no |
| Urinary disorders | 1. yes 2.no |
| Other chronic diseases | 1. yes 2.no |
