# Supplementary material for: Health-related quality of life and long-term care needs among elderly individuals living alone: a cross-sectional study in rural areas of Shaanxi Province, China
Source: BMC Public Health. 2013 Apr 8;13:313. doi: 10.1186/1471-2458-13-313 (PMC3642010; doi:10.1186/1471-2458-13-313)
Supplement: Additional file 2 — The Medical Outcomes Study 36-Item Short-Form Health Survey. [file 1471-2458-13-313-S2.doc]

## Additional File 2:The Medical Outcomes Study 36-Item Short-Form Health Survey

| 1. In general, would you say your health is: |  |
| --- | --- |
| Excellent | 1 |
| Very good | 2 |
| Good | 3 |
| Fair | 4 |
| Poor | 5 |

| 2. Compared to one year ago, how would your rate your health in general now? |  |
| --- | --- |
| Much better now than one year ago | 1 |
| Somewhat better now than one year ago | 2 |
| About the same | 3 |
| Somewhat worse now than one year ago | 4 |
| Much worse now than one year ago | 5 |

The following items are about activities you might do during a typical day. Does your health now limit you in these activities? If so, how much?

(Circle One Number on Each Line)

|  | Yes, Limited a Lot | Yes, Limited a Little | No, Not limited at All |
| --- | --- | --- | --- |
| 3. Vigorous activities, such as running, lifting heavy objects, participating in strenuous sports | 1 | 2 | 3 |
| 4. Moderate activities, such as moving a table, pushing a vacuum cleaner, bowling, or playing golf | 1 | 2 | 3 |
| 5. Lifting or carrying groceries | 1 | 2 | 3 |
| 6. Climbing several flights of stairs | 1 | 2 | 3 |
| 7. Climbing one flight of stairs | 1 | 2 | 3 |
| 8. Bending, kneeling, or stooping | 1 | 2 | 3 |
| 9. Walking more than a mile | 1 | 2 | 3 |
| 10. Walking several blocks | 1 | 2 | 3 |
| 11. Walking one block | 1 | 2 | 3 |
| 12. Bathing or dressing yourself | 1 | 2 | 3 |

During the past 4 weeks, have you had any of the following problems with your work or other regular daily activities as a result of your physical health?

(Circle One Number on Each Line)

|  | Yes | No |
| --- | --- | --- |
| 13. Cut down the amount of time you spent on work or other activities | 1 | 2 |
| 14. Accomplished less than you would like | 1 | 2 |
| 15. Were limited in the kind of work or other activities | 1 | 2 |
| 16. Had difficulty performing the work or other activities (for example, it took extra effort) | 1 | 2 |

During the past 4 weeks, have you had any of the following problems with your work or other regular daily activities as a result of any emotional problems (such as feeling depressed or anxious)?

(Circle One Number on Each Line)

|  | Yes | No |
| --- | --- | --- |
| 17. Cut down the amount of time you spent on work or other activities | 1 | 2 |
| 18. Accomplished less than you would like | 1 | 2 |
| 19. Didn't do work or other activities as carefully as usual | 1 | 2 |

20. During the past 4 weeks, to what extent has your physical health or emotional problems interfered with your normal social activities with family, friends, neighbors, or groups?

(Circle One Number)

| Not | 1 |
| --- | --- |
| Slightly | 2 |
| Moderately | 3 |
| Quite | 4 |
| Extremely | 5 |

21. How much bodily pain have you had during the past 4 weeks?

(Circle One Number)

| None | 1 |
| --- | --- |
| Very mild | 2 |
| Mild | 3 |
| Moderate | 4 |
| Severe | 5 |
| Very severe | 6 |

22. During the past 4 weeks, how much did pain interfere with your normal work (including both work outside the home and housework)?

(Circle One Number)

| Not at all | 1 |
| --- | --- |
| A little bit | 2 |
| Moderately | 3 |
| Quite a bit | 4 |
| Extremely | 5 |

These questions are about how you feel and how things have been with you during the past 4 weeks. For each question, please give the one answer that comes closest to the way you have been feeling.

How much of the time during the past 4 weeks . . .

(Circle One Number on Each Line)

|  | All of the Time | Most of the Time | A Good Bit of the Time | Some of the Time | A Little of the Time | None of the Time |
| --- | --- | --- | --- | --- | --- | --- |
| 23.Did you feel full of pep? | 1 | 2 | 3 | 4 | 5 | 6 |
| 24.Have you been a very nervous person? | 1 | 2 | 3 | 4 | 5 | 6 |
| 25.Have you felt so down in the dumps that nothing could cheer you up? | 1 | 2 | 3 | 4 | 5 | 6 |
| 26.Have you felt calm and peaceful? | 1 | 2 | 3 | 4 | 5 | 6 |
| 27.Did you have a lot of energy? | 1 | 2 | 3 | 4 | 5 | 6 |
| 28.Have you felt downhearted and blue? | 1 | 2 | 3 | 4 | 5 | 6 |
| 29. Did you feel worn out? | 1 | 2 | 3 | 4 | 5 | 6 |
| 30.Have you been a happy person? | 1 | 2 | 3 | 4 | 5 | 6 |
| 31.Did you feel tired? | 1 | 2 | 3 | 4 | 5 | 6 |

32. During the past 4 weeks, how much of the time has your physical health or emotional problems interfered with your social activities (like visiting with friends, relatives, etc.)?

(Circle One Number)

| All of the time | 1 |
| --- | --- |
| Most of the time | 2 |
| Some of the time | 3 |
| A little of the time | 4 |
| None of the time | 5 |

How TRUE or FALSE is each of the following statements for you.

(Circle One Number on Each Line)

|  | Definitely True | Mostly True | Don't Know | Mostly False | Definitely False |
| --- | --- | --- | --- | --- | --- |
| 33.I seem to get sick a little easier than other people | 1 | 2 | 3 | 4 | 5 |
| 34.I am as healthy as anybody I know | 1 | 2 | 3 | 4 | 5 |
| 35.I expect my health to get worse | 1 | 2 | 3 | 4 | 5 |
| 36. My health is excellent | 1 | 2 | 3 | 4 | 5 |
