# Supplementary material for: Health-related quality of life and long-term care needs among elderly individuals living alone: a cross-sectional study in rural areas of Shaanxi Province, China
Source: BMC Public Health. 2013 Apr 8;13:313. doi: 10.1186/1471-2458-13-313 (PMC3642010; doi:10.1186/1471-2458-13-313)
Supplement: Additional file 3 — Long-term care needs among elderly living alone. [file 1471-2458-13-313-S3.doc]

## Additional File 3:Long-term care needs among elderly living alone

1. Health knowledge: (Whether the individuals needed the following knowledge: how balanced diet, reasonable sleep regulation patterns of life, prevention and treatment of a variety of common chronic diseases, the family of rational drug use): 1. yes 2. no.

2. Whether the individuals needed hospital-based care. (a simple wound care, oxygen inhalation, intramuscular, intravenous infusion, enema, catheterization): 1. yes 2. no.

3. Whether the individuals needed of long-term care needs: (whether in urgent need of the following care: oral care, pressure ulcer care, shampoo bed, perineal scrub and warm bath): 1. yes 2. no.

4. Nursing home care: 1. yes 2. no.

5. Whether the individuals needed of community-based geriatric care: 1. yes 2. no.
